# Microbial Nanoparticles in Biological Plant Protection

**DOI:** 10.3390/ijms26062492

**Published:** 2025-03-11

**Authors:** Tomasz Maciag, Edmund Kozieł, Małgorzata Dudkiewicz, Katarzyna Otulak-Kozieł

**Affiliations:** 1 Department of Botany, Institute of Biology, Warsaw University of Life Sciences—SGGW, Nowoursynowska Street 159, 02-776 Warsaw, Poland; katarzyna_otulak@sggw.edu.pl; 2 Department of Biochemistry and Microbiology, Warsaw University of Life Sciences—SGGW, Nowoursynowska Street 159, 02-776 Warsaw, Poland; malgorzata_dudkiewicz@sggw.edu.pl

**Keywords:** nanotechnology, plant protection, plant disease, green synthesis

## Abstract

Nanoparticles are small structures that differ in terms of their shape and composition; their high surface-to-volume ratio is responsible for their unique properties that make them perfect mediators for the delivery of substances. Nanoparticles do not only include metallic spheres but also complex polysaccharides capsule viruses or bacterial protein complexes (which can be considered bionanoparticles), which are 1–100 nm in size. Although nanoparticles are most widely studied from medical perspectives, their potential applications are almost limitless. One such promising use of functional nanoparticles is for plant protection against diseases. Although the precise use of nanoparticles decreases the need for the use of other chemical compounds, thanks to their increased product stability and delivery to a target site, the production of nanoparticles is often burdened by large quantities of toxic wastes. This problem can be limited if we apply the bioreactor green synthesis method, which includes the production of nanoparticles with the use of microorganisms. Bacteria can produce nanoparticles internally, externally, by only producing metabolites used for nanoparticle production directly, e.g., polysaccharides or surfactants, or indirectly as reducing agents for metal nanoparticle production. Regardless of the source of the nanoparticles, they can be widely used in processes from plant disease/pathogen detection to disease suppression. The endless variety of materials for nanoparticle production and the possible modifications that nanoparticles can be subjected to makes it impossible to predict how their structures will be used in the future. Nevertheless, in this study, we would like to turn attention to the fact that although nanoparticles are viewed as synthetic structures, they are ever-present in the microbial world and play an important part in intermicrobial interactions. As nanoparticle usefulness has been tested over years of co-evolution, it may be useful to look for potential future directions for this fascinating technology.

## 1. Introduction

Nanoparticles are small 1–100 nm structures with a high surface-to-volume ratio [1]. The large surface area makes this material ideal for numerous biological applications and was first investigated as a carrier of active substances, especially for antibodies, and produced through a process called micelle polymerization [2]. Today, synthetic silver, gold, and copper nanoparticles are intensively studied and are the most commonly used materials in commercial products [3]. Although metallic nanoparticles, particularly gold nanoparticles have been produced and used by humans from ancient times, primarily by the Romans for staining glass [4], their application range greatly widened from the late XX century, when studies on the medical use of nanoparticles began [5]. Today, nanoparticles are produced by a wide array of methods for numerous applications [6]; these methods can be divided into top-down and bottom-up approaches [1]. The top-down approach relies mainly on physical methods for breaking down large structures into nanostructures. However, this relatively simple approach often yields nanoparticles with surface structure imperfections. On the other hand, the bottom-up approach relies on creating nanostructures from smaller building blocks, for example, in chemical or microbial synthesis. This approach usually produces more uniform nanoparticles and is often relatively cost effective [7].

The commonly used bottom-up nanoparticle synthesis techniques often produce a large amount of toxic waste depending on the used method and the utilized materials. This could be a huge disadvantage of the nanoparticles that can play a huge role in the transformation to more sustainable agriculture [8]. Therefore, it is imperative to use fewer toxic ingredients for nanoparticle production to both decrease the negative effect of produced wastes and decrease the nanoparticle toxicity [9]. This ambitious goal can be achieved by the use of plant extract containing cheap and biodegradable reducing agents, which not only reduce the cost of nanoparticle production and the amount of toxic waste but can also incorporate biologically active plant metabolites [9].

However, the promising green synthesis of nanoparticles is not without drawbacks. The major disadvantage of green synthesis is that information on the mechanism behind it is still missing. Biological processes that usually yield products with limited amounts of toxic wastes are less controllable, and complex structures, such as nanocapsules or mesoporous nanoparticles, cannot yet be created with that approach. The relatively most commonly used method for green synthesis of nanoparticles uses plant extracts, which are easily obtainable, and the mechanisms of this process are relatively well-studied [10].

The efficiency and simplicity of nanoparticle production using plant extracts make this method not only more sustainable but also cost-efficient [11]. Therefore, many new research articles have been published describing multiple applications of nanoparticles produced using this method [11]. The versatile role of plant metabolites during nanoparticle synthesis results in a huge reduction of the additional components needed for nanoparticle synthesis; therefore, the plants used for such purposes are called “plant bioreactors” [12]. For example, the extract of *Lawsonia inermis* L. can be used as both a reducing and stabilizing agent to prepare the 46 ± 5 nm silver nanoparticles for effective dye degradation [13].

Furthermore, fungi can be used for the biological production of nanoparticles [14,15]. Filamentous fungi can withstand toxic waste pollution, which is attributed inter alia to the extensive production of extracellular enzymes. This makes them perfect factories of nanoparticle production from heavy metals [14]. Their metabolic activity leads to the reduction and aggregation of heavy metals into less toxic nanoparticles.

Another promising group of microorganisms is microalgae [16]. This diverse group of organisms combines the advantage of plant metabolisms due to the ability to perform photosynthesis microalga and is forced to produce a wide range of metabolites useful for nanoparticle production. On the other hand, their unicellular structure makes these organisms perfect for production in bioreactors in heavily controlled conditions. The use of microalgae is especially prominent in wastewater treatment, where they can play a versatile role in the production of nanosensors through bioremediation [17].

Furthermore, bacteria can be used to create nanoparticles. The parameters of the liquid culture of unicellular organisms, such as bacteria, can be easily controlled and adjusted to the particular needs of the process, making microbial-origin nanoparticle production economically efficient [9]. Unfortunately, the enzymes responsible for the creation of nanoparticles are often unknown.

Another promising group of organisms that can be used for the creation of bionanoparticles is viruses. Viruses, upon infection of the host cells, use the host protein synthesis machinery to produce large amounts of proteins, which presents the ability to self-assemble into nanostructures used by the viruses to protect their genome from the external environment [9]. These proteins can be chemically modified and used as carriers for numerous biologically active chemicals [16]. Viral capsids can be produced even without the presence of the virus when the host is transformed with the genes responsible for the production of capsid-building proteins. These structures, produced by hosts lacking the genetic material of the virus, are called viral-like particles and find wide application in medical approaches, for example, during the production of vaccines [17]. A good example of the application of viral-like particles is the Novavax vaccine against SARS-CoV-2 [18]. The production of viral proteins devoid of viral genome material is not a human invention. Some viruses, e.g., hepatitis B virus (HBV), during viral particle assembly produce a large proportion (c.a. 90%) of empty capsids devoid of the viral genome [19]. The study of the viral particle assembly has led to the development of systems for the production of viral proteins that self-assemble themselves into empty capsids called viral-like particles (VLPs) [20]. These VLPs produced in vitro lack the infective properties of a parent virus and can be used as vaccines, tools for gene therapy, or new promising nanomaterials [21]. On the other hand, viral infections lead to host genome modification by the incorporation of the genes of viral origin into the host chromosome, called endogenous viral elements (EVEs) [22]. In bacteria, the VI secretion system and tailocins are examples of such EVEs that contribute positively to the host fitness [23]. These structures are built by bacteriophage-inherited self-assembling proteins. The VI secretion system consists of sets of proteins assembling into a tubular polymer building sheath and membrane complex. After attachment to a target cell, the sheath releases the energy, pushing the spike into the target cell and releasing the effectors into the target cell cytoplasm during the injection-like process. The proteins building up the elements of T6SS are structurally homologous to the bacteriophage’s tail proteins, suggesting the bacteriophage origin of this system [24].

The tailocins are produced by many gammaproteobacteria, and their production is induced by stress factors. The tailocins are transported to the cell pool and released during cell lysis. The tailocins produced by one strain show bacteriocidal capabilities against closely related strains but not against the producer [25]. This makes them good candidates for metabolites targeted against important bacterial pathogens and possible scaffolds to produce new, more specific antimicrobials [26].

Nanoparticles have a wide range of applications in current technologies, from chemical synthesis to biomedical applications [1]. However, due to the implementation of green synthesis methods, the range of applications can largely extend thanks to the decreased cost of production [9]. This can cause the rapid development of nanotechnologies in agriculture, which can help to address the growing food demand. In this review, we aim to identify the main ways in which the nanoparticles can be used to protect plants from their diseases and how these nanoparticles can be produced with the use of microorganisms. Although nanotechnology can lead to the development of unpredicted new technologies, the future for nanotechnologies in the prevention of diseases in agriculture lies within the use of nanosensors for the effective and sensitive detection of pathogens, bactericidal properties of metallic nanoparticles for sterilisation and antimicrobial surface coatings, nanoencapsulation for triggered release of effector substances, and also in the use of target-specific protein-based nanoparticles [1].

**Figure 1:**
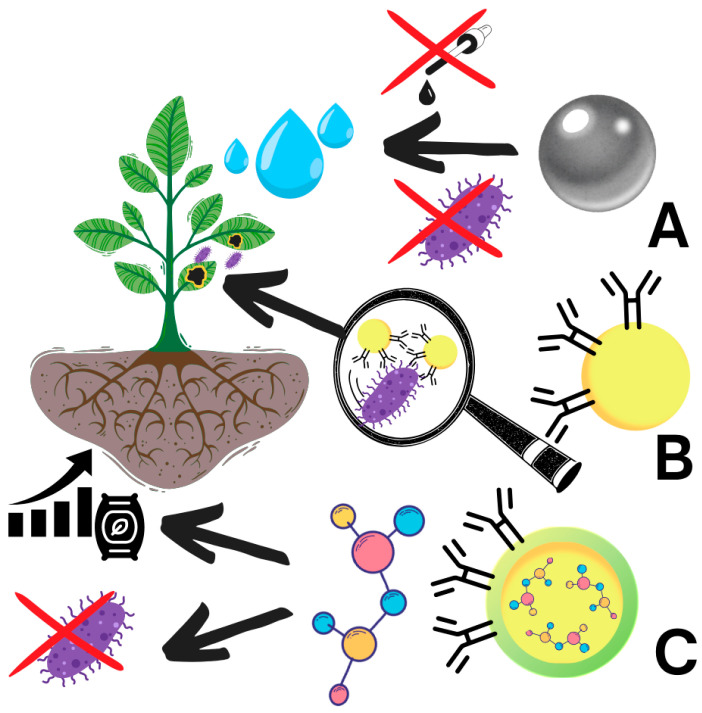
Schematic representation of nanoparticle application in agriculture. (**A**): Nanoparticles can be used to remove (X) water contamination, including pathogens and harmful chemicals, including dyes; (**B**) be used to detect important plant pathogens; (**C**) for the targeted delivery of chemicals to more efficiently stimulate plant growth and suppress the pathogens.

Nanoparticles are a promising technology that finds a wide range of applications in more and more aspects of the economy [27]. Their surface-to-volume ratio makes this structure an effective delivery system for active substances, e.g., in medicine, but also makes them perfect candidates for the development of sensitive and robust sensors [1]. Nanoparticles are still, however, mostly viewed as a small, sophisticated technology preserved for complex applications. However, thanks to green synthesis, their production becomes more economically feasible and largely reduces the toxicity of waste products [28]. Therefore, we observe the growing trend in research dedicated to the use of nanoparticles in agriculture. Currently, nanoparticles can be used to clear the water [29] from plant pathogens and harmful chemicals, including dyes [30,31], detect important plant pathogens [32], and serve as delivery vehicles for the targeted release of chemicals to stimulate plant growth [33] or suppress disease [34] (Figure 1). In this article, we would like to promote new research on the mechanisms of nanoparticle green synthesis, which we believe will facilitate the use of this method, make them more economically feasible, and decrease their negative impact on the environment.

## 2. Nanosensors

The first line of protection of plants against diseases is the early detection of pathogens. When the disease is detected at the early stage of infection, the losses can be largely mitigated by the isolation of healthy plants or by applying plant-protective agents [35]. On the other hand, when the disease spreads to the whole field, even manageable diseases can cause huge losses in crop yield and quality [36]. Unfortunately, most methods of exact pathogen identification are costly, time-consuming, and can lack sensitivity at the early stages of infection. What is more, the early stage of infection rarely causes recognisable or even visible symptoms, which makes the potential selection of the disease detection method even more challenging [35]. Therefore, it is recommended to screen source plants and further the plant material at crucial points of development for the presence of a wide range of pathogens to avoid later problems [37]. Moreover, global climate change has created a situation where the range of pathogens infecting economically important crops is constantly growing in different parts of the world [38]. Therefore, there is a constant need for the development of new, reliable, and easy methods for the quick identification of the most pressing pathogens to limit their spread, which can be partially achieved with the use of nanoparticles [35].

Biogenic nanoparticles (nanoparticles created with the use of biological materials) can serve as a perfect vessel for the attachment of DNA probes, antibodies, or colorimetric tests, creating sensitive nanosensors [39]. The nanomaterials created with the use of microorganisms can be chemically modified to present specific antibodies or DNA fragments specific to a certain pathogen or its toxin [39]. The attachment of the probes to the nanoparticles increases the probe’s long-term stability and increases the detection specificity (due to the presence of multiple antibodies and DNA fragments on one nanoparticle), but the physicochemical parameters of the nanoparticles can also be used for pathogen detection [40].

**Figure 2:**
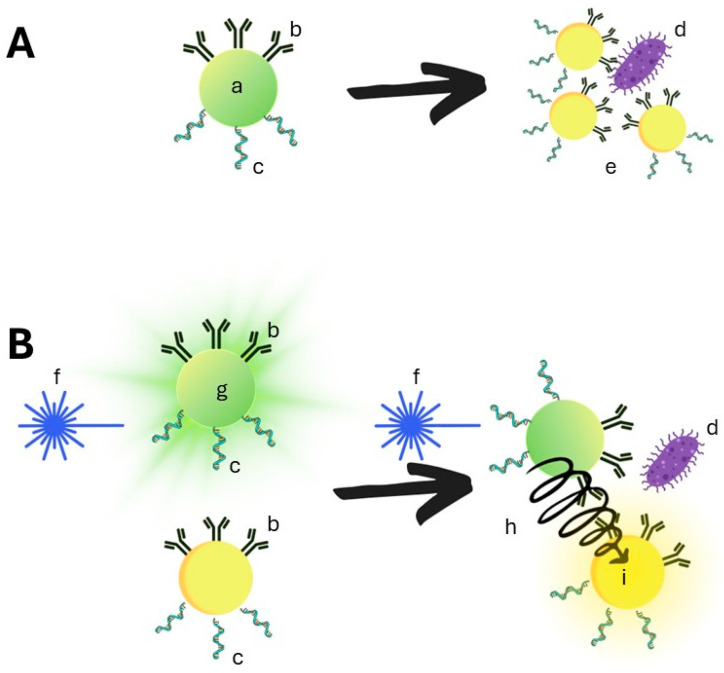
Schematic representation of nanosensors. (**A**) based on colorimetric biosensors: (**a**): non-aggregated nanoparticles are visible in visible light, and the nanoparticles can be modified with antibodies (**b**) or nucleic acid (**c**). After the addition of the antigen, the nanoparticles aggregate around the antigen (**d**), which causes the change of the nanoparticle’s visual color (**e**). (**B**) Quantum dots nanoparticles use fluorescent material that can be excited by blue light (**f**). The excited Quantum dots emit the light of a certain wavelength, when different Quantum dots differing in the excitation wavelength and the emitted light are brought into proximity by binding to one antigen (**d**), the energy from one QD can be non-fluorescently transmitted to another (**h**), bringing it to an excited state and causing the emitting of light of different wavelengths (**i**).

Colorimetric biosensors rely on the nanoparticle’s ability to change colour depending on the nanoparticle size. The antibodies attached to the nanoparticles bind the antigen on the surface of the pathogen, forming a larger complex and leading to the change of colour (Figure 2A). Several biogenic nanoparticles have been created based on gold nanoparticles, which can facilitate the detection of, e.g., *Potato virus X* (PVX) [41] or *Pantoea stewartia* [42] (Table 1). The colorimetric approach gives fast and reliable results; however, it lacks sensitivity for the early detection of pathogens due to the necessity to create a visible effect. Therefore, other more sophisticated detection approaches are more applicable for early detection, although the simplicity and speed of detection make it a good candidate for developing methods for pathogen identification on symptomatic yet hard-to-recognise plant material [43]. Currently, there are no available microbial-origin nanoparticles that have been developed to detect plant pathogens in the colorimetric approach; many bacterial species can be used to create gold nanoparticles suitable for this approach, including *Escherichia*, *Lactobacillus*, *Pseudomonas*, *Rhodococcus*, *Rhodopseudomonas*, *Shewanella*, *Thermomonospora*, and possibly many other bacteria genera [44].

The alternative method of optical detection with higher sensitivity is the use of fluorescence. The nanoparticles can be either fluorescently labelled by the chemical modification of the nanoparticle surface or produced from semiconductor nanocrystals with innate fluorescence properties (Table 1) [52]. Exciting fluorochrome with a specific wavelength of light results in strong fluorescence in a shorter wavelength spectrum, decreasing the background noise. However, most of the nanoparticles are not fluorescent and require fluorescent labelling to use that method of detection. The golden nanoparticle, however, increases the signal thanks to their ability to be good electron acceptors. The fluorescent-labelled golden nanoparticles are not used for quantification but for localization [53]. The quantitative methods using fluorescently labelled nanoparticles rely on the phenomenon called fluorescence resonance energy transfer (FRET) [53]. FRET is a phenomenon that relies on the fact that if two chromophores are close to each other, the electron from the excited one (donor) can jump to the other (acceptor) without emitting light, exciting the donor molecule [54] (Figure 2B). This phenomenon can be used on golden nanoparticles to quantify telomerase inside living cells. A golden nanoparticle is chemically modified with a hairpin forming a DNA fragment and the non-covalently bound second strand of DNA tagged with chromophores at both ends of the sequence. In this state, the donor and acceptor chromophores are distant from each other, and no energy transfer occurs. When the telomerase unwinds the double-stranded DNA, the hairpins are formed, and the chromophores decrease distance to each other, enabling FRET. Then, the donor chromophore is excited, and the fluorescence of both the donor and acceptor is measured, enabling the measurement of the telomerase quantity [55].

For the detection and quantification of plant pathogens with the use of the FRET phenomenon, the different types of nanoparticles are called Quantum dots (Qdots, QD). These semiconductive luminescent nanocrystals have several advantages, making them perfect for FRET applications [56]. The luminescence wavelength of the QD is positively correlated with the nanocrystal size and is much less prone to light bleaching than traditional chromophores. Therefore, the fluorescence wavelength of the QDs can be adjusted with the size and have much better stability than traditional chromophores [43]. Additionally, this type of nanoparticle is easily created by multiple species of microorganisms, which is especially interesting since the creation of QDs with physicochemical methods is much more expensive [57]. The bacteria genera used for the production of QD include *Acidothiobacillus* [58], *Bacillus* [59], *Escherichia* [60], *Exiguobacterium* [61], *Halobacillus* [62], *Pedobacter* [63], *Pseudomonas* [64], *Raoutella* [65], *Rhodopseudomonas* [66], *Serratia* [59,60], and *Stenotrophomonas* [67], and fungal genera include *Aspergillus* [68], *Candida* [69], *Coriolus* [70], *Fusarium* [71], *Helminthosporium* [72], *Penicillium* [73], *Phanerochaete* [74], *Phomopsis* [75], *Pleurotus* [76], *Rhizopus* [77], *Saccharomyces* [78], *Trichoderma* [79], and *Trametes* [57,62]. As the QD is most commonly used for nucleotide analysis, the method for the detection of plant pathogens relies mainly on the detection of their DNA; for example, Bakhori et al., 2013 presented the Cadmium Selenide QD tagged with ssDNA with a Cy5-added FRET acceptor to detect *Ganoderma boninense*, a fungal pathogen, causing basal stem rot [47]. On the other hand, Rad et al., 2012 added a tioglicilic acid moiety to cadmium telluride QD and tagged them with an antibody targeted against an immunodominant membrane protein IMP of Candidatus *Phytoplasma aurantifolia*, thus constructing a biosensor for detecting the causing agent of witches broom disease in lime [48]. Shojaei et al. developed a cadmium-telluride quantum dots-based system for Citrus tristeza virus (CTV) detection with the use of antibodies targeted against coat protein, which allowed for viral particle detection from 220 ng/mL [49]. On the other hand, Safarnejad et al. developed a similar Quantum dot detection system based on the detection of CTV coat protein with antibodies attached to telluride quantum dots and used two different detection systems. The first was based on FRET, where the energy was transferred from quantum dot to rhodamine. The second approach used the fact that aggregation by binding to the antigens Quantum dots emits stronger signals. The detection limit of both methods was 198 ng/mL and 246 ng/mL of purified CTV-CP [50]. Safarpour et al. proposed a QD-based strategy to protect plants against Beet necrotic yellow vein virus BNYVV through the detection of its major vector *Polymyxa betae* (Keskin) thanks to tioglicolic acid-modified cadmium-telluride quantum dots tagged with antibodies targeted against the vector’s glutathione-S-transferase [51]. Majumder et al. used cadmium selenide QDs tagged with antibodies directed against the coat protein of banana bunchy top virus BBTV to increase 2.5-fold the detection limit of electrochemical ELISA detection [80] (Table 1).

Although we still do not have many methods for plant pathogen detection using nanoparticles, the possibilities are almost limitless. A huge advantage of the use of nanoparticles in the detection of plant diseases lies within the much smaller quantity detection limit, allowing relatively quick identification of pathogens in diluted samples, which is crucial for early detection and stopping the spread of the disease and pathogen propagation [81]. Nanoparticles, on the one hand, can be coupled with highly specific antibody or molecular-associated methods, such as ELISA, PCR, LAMP, or plasmon resonance, further increasing their sensitivity and specificity, but can also increase the robustness of detection methods for in-field detection [81,82]. These properties have led to the development of several interesting and sensitive methods for plant pathogen detection, e.g., *Citrus tristeza virus* (CTV) detection by Qdots (QD) tagged with antibodies targeted against its coat protein allows for the detection of 0.13 μg mL^−1^, which is 10 times higher than the standard ELISA method with higher sensitivity, 93% vs. 80%, and specificity, 94% vs. 88% [42]. Nanoparticles, on the other hand, can be used to develop not only highly sensitive laboratory methods with higher-than-standard method detection limits but also help to develop robust and high-throughput detection systems for the farmers to easily assess their crops in case of suspicious symptoms [82] with the possibility of checking a higher amount of samples. The perfect example of such a promising method for a quick in-field detection method is the gold nanoparticle-based immunochromatographic test strip for the detection of corn pathogen *Pantoea stewartii* subsp. *stewartii*, from 1 × 10^5^ cfu/mL in 10 min with detected cross-reactivity [83].

Metallic nanoparticles are not only the most commonly produced nanoparticles [3] that can be used as carriers for the development of nanosensors of important plant pathogens [1], but also possess bacteriocidal properties, which can be used to protect plant seed material from infections or disinfect agricultural equipment (especially if the effect of antibacterial and antifungal activity is relatively long-term) [84]. Therefore, nanoparticles could be added to the synthetic additional seed dressing/coat or cover the natural seed coat during the nano-priming process [85]. The nanomaterials used for seed coatings can reduce the amount of chemicals needed for seed coatings and promote plant development by the controlled release of plant hormones and nutrients and physically and chemically protect emerging plants from pathogen invasion [86].

## 3. Metallic Nanoparticles

Although silver nanoparticles are most widely used for their antimicrobial potential [87], other metals such as gold [4], copper [87], selen [88], nickel [89], zinc [90], titanium [91], and iron oxide [91] also possess confirmed antimicrobial properties [92]. This variety of materials can help find the proper material for the target application.

### 3.1. Bacteriocidal Nanoparticles

Silver nanoparticles are the most widely studied nanoparticles due to their antimicrobial, antioxidant, and anti-inflammatory properties and their low toxicity to plants and animals [86]. Although the production of silver nanoparticles consumes relatively costly silver, the production of these nanoparticles is relatively easy, and there are multiple available methods for silver nanoparticles’ green synthesis, mainly based on plant extracts [85]. Plant extracts not only reduce the consumption of toxic chemicals, but the biologically active plant secondary metabolites can also be incorporated into the synthesised nanoparticles, which can further increase their antimicrobial properties. The synergy of the antimicrobial activity of silver nanoparticles can be achieved due to the nanoparticle-mediated cell membrane damage, which can enhance the activity of antimicrobial plant secondary metabolites [93]. It has been shown that three out of five tested plant secondary metabolites, 3-chloroplumbagin, plumbagin, and ramentaceone, synergistically act with silver nanoparticles against the bacterial pathogen *Staphylococcus aureus*, thanks to the membrane permeabilization by silver nanoparticles [94]. Silver nanoparticles synthesised with *Piper nigrum* L. leaf or stem extract show antimicrobial properties against plant pathogens: *Citrobacter freundii* and *Erwinia cacticida* show synergy when combined with the antibiotic Chloramphenicol [95]. However, plant extract can not only be used for the green synthesis of silver nanoparticles against plant diseases.

For example, the culture supernatant from the culture of *Pseudomonas rhodesiae*, a plant-beneficial microorganism, can be used to produce silver NPs against the soft rot pathogen *Dickeya dadantii* (Table 2) [96]. Furthermore, silver-resistant *Bacillus cereus* supernatant shows plant-protective properties against *Xanthomonas oryzae*, an economically important pathogen of rice *Oryza sativa* L. [97]. The list of microorganisms that produce antimicrobial particles is ever-extending [98]. Nanoparticles produced with microorganisms are obtained due to chemical reduction [99] by microbial metabolites or by enzymatic synthesis [100]. This process can take place both inside and outside of the bacterial cell. However, to obtain pure and non-aggregated NPs in the most controllable process, NPs are most commonly produced using microbial supernatants [98].

Furthermore, other materials can be used to produce nanoparticles protecting plants from pathogens (Table 2) [92]. Gold nanoparticles produced extracellularly by the plant-beneficial strain of fungi *Trichoderma hamatum* possess bacteriocidal properties against pathogenetic bacteria: *Bacillus subtilis*, *Staphylococcus aureus*, *Pseudomonas aeruginosa*, and *Serratia* sp. [112]. In addition, copper nanoparticles have wide potential for plant protection against microbial pathogens such as: *Agrobacterium tumefaciens*, *Dickeya dadantii*, *Erwinia amylovora*, *Pectobacterium carotovorum*, *Pseudomonas corrugata*, *Pseudomonas savastanoi* pv. savastanoi, and *Xanthomonas campestris* pv. Campestris, due to the known copper antimicrobial properties. The nanoparticle form of application in this case increases the activity of applied copper, reducing the toxic effect of adding copper to the environment [113]. The copper nanoparticles can be produced biologically, for example, by non-pathogenic bacteria: *Pseudomonas stutzeri* increasing the sustainability of this solution [87].

Nickel and nickel nanoparticles can have adverse effects on plant biology. On the one hand, it is an essential microelement; on the other hand, it can be toxic to plants in high concentrations. The toxicity of nickel and its nanoparticles can be largely reduced by the exogenous application of plant hormones or silicone [89]. These nanoparticles can be produced with the use of nickel-resistant bacterium *Microbacterium* sp. MRS-1 [114]. Nevertheless, the application of nickel nanoparticles in agriculture is largely limited due to their toxicity to plants [89]. Zinc and its nanoparticles, similar to nickel, can have adverse effects on plants. On the one hand, its important microelements can lead to oxidative stress and cell damage. It has been shown that various microorganisms, such as *Aspergillus fumigatus* [115], *Aspergillus niger* [116], *Cyanobacterium Nostoc* [117], *Halomonas elongate* [118], *Pichia fermentas* [119], *Pichia kudriavzevii* [120], and *Staphylococcus aureus* [121], produce zinc nanoparticles with antimicrobial properties [90].

Although there have been several discoveries of microorganisms producing titanium-based nanoparticles with antimicrobial properties, including organisms such as *Aeromonas hydrophila* [122] and *Bacillus mycoides* [123,124], not much progress has been made regarding their agricultural applications. This might be partially attributed to the fact that the influence of titanium nanoparticles on plants is largely understudied, and the data collected so far indicate their negative influence on the growth of soybean (*Glycine max.* L.) [91].

One of the most promising materials for the construction of nanoparticles is iron oxide, thanks to its super magnetic properties, which are widely studied for medical applications [125]. Although magnetic nanoparticles are mainly synthetically produced, they appear in all living organisms and are commonly found in bacterial magnetosomes—structures used by microorganisms, e.g., *Magnetospirillum magnetotacticum*, to operate in Earth’s magnetic field. The microbial magnetic nanoparticles are more stable and uniform, and they can be acquired by a synthetic approach, making them perfect candidates for biotechnological applications [126].

### 3.2. Nanofertilizers

Some of the elements used for the production of nanoparticles are important plant microelements, e.g., iron, manganese, zinc, boron, copper, molybdenum, selenium, and silicone [127]. These elements need to be supplemented in intensive farming; however, the fertilisers in soil application lead to fertilisers escaping into the environment, which not only increases the cost of fertilisation but also causes severe environmental pollution. Therefore, the foliar application of nanoparticle-based fertilisers seems to be a perfect method for microelement fertilisation [127].

For example, selenium nanoparticles can find their use in agriculture mostly due to the plant growth promotion properties, as well as the reduction of oxidative stress coupled with antimicrobial properties [128]. Currently, selenium nanoparticles are more widely studied as plant growth stimulants, but the activity is indirect, since selenium is a nonessential element for plants, and part of its beneficial properties can be attributed to the suppression of plant pathogens [129]. Selenium nanoparticles can be produced by microorganisms isolated from soils polluted with this element, e.g., *Thauera selenatis* [129]. It has been proven that *Thauera selenatis* can produce and secrete 150 nm diameter selenium nanoparticles with potential for industrial applications [130].

### 3.3. Water Purification

One of the most important aspects of agriculture is proper irrigation, which is essential for proper crop growth. However, the water used for irrigation can carry harmful chemicals or be a source of many important plant pathogens, especially in treated wastewater [131]. Wastewater treatment removes major pollutants from the water, but some residual contaminants, especially heavy metals and biological contaminants, are difficult to fully remove from the water using traditional water sanitation methods. However, nanotechnology can largely help to solve this issue. There are numerous new nanomaterials for wastewater treatment, which have found use in water treatment in agricultural applications, which have been reviewed by Kuhn et al., 2022 [132].

For example, silver nanoparticles synthesised by microalgae can purify the water for agricultural applications [29,133]. The nanoparticles synthesised by microorganisms can also be used to remove the important pathogenic bacteria from irrigation water, preventing the spread of important diseases and food contamination [34]. Silver nanoparticles can also help to remove the important chemical contaminants, such as dyes [30]. For example, Brilliant Blue R dye is used in textile staining due to its stability and has a long half-life in the environment. Hopefully the nanoparticle produced with *Chlorella vulgaris* can stimulate the photodegradation of this contaminant [31].

## 4. Viral Nanoparticles

Various viruses have been mainly considered a threat to human and animal health and plant food supply security. However, their neatly organised nanostructures can have a wide range of applications in medicine but also in agriculture. Thanks to the advancement in molecular biology methods, the modification of the capsid structure is facilitated, and today, viral capsids have a wide range of applications in modern medicine and show huge potential in industry and farming [134]. Plant viruses can serve as a perfect tool for plant genome modification not only thanks to the delivery system but also as a source of potent primers for exogenous gene expression [135]. Plant viruses can also be used for the detection or imaging of targeted antigens or as vehicles for vaccines since they are considered safe for humans [134]. An interesting example can be the application of modified *Potato virus X* (PVX) overproduced in wild tobacco *Nicotiana clevelandii* for the detection of diuron herbicide [136]. PVX can also be used to transform *Nicotiana benthamiana* to produce a yeast killer toxin (KT), which is active against important plant pathogens: *Pseudomonas syringae*, *Erwinia carotovora*, *Botrytis cinerea*, and *Fusarium oxysporum* [137].

However, viruses with the widest potential to protect plants against diseases seem to be bacteriophages. These viruses, by nature, can infect plant pathogenic bacteria, thus largely limiting their populations [138]. Bacteriophage nanostructure has evolved to target different bacteria species, leading to their lysis. The huge advantage of applying bacteriophages to control diseases in their specificity is that they can help reduce the number of pathogens without a negative impact on local microbial diversity and without risk to other organism populations. Plant pathogens that can be largely controlled with bacteriophages include *Acidovorax citrulli* [139], *Clavibacter michiganensis* [140], *Dickeya solani* [141], *Erwinia amylovora* [142], *Pectobacterium atrosepticum* [143], *Pectobacterium parmentieri* [144], *Pseudomonas syringae* [145], *Ralstonia solanacearum* [146], *Xanthomonas citri* [147], *Xanthomonas euvesicatoriae* [148], and *Xanthomonas oryze* [138,149]. Unfortunately, although there are numerous studies involving the potential use of bacteriophages for the control of plant diseases, the number of physical products available on the market ready to use is largely limited [138]. On the other hand, bacteriophages can be used to develop early pathogen detection methods. The ability of bacteriophages to reproduce in bacteria, their uniformity, and the tools of molecular biology make bacteriophages ideal candidates for developing modern nanoparticle-based plant pathogen detection methods [138]. Bacteriophages infecting hosts can have two different life cycles, lytic and lysogenic. In the lytic cycle, after the virus’s genetic material is injected into the bacterial host, the virus uses the host machinery to produce the viral proteins, including capsid proteins, and replicates its genome. The new viruses are assembled inside the cell, which eventually bursts, releasing new phages. Sometimes the bacteriophages can use a lysogenic cycle and enter the host and incorporate their genetic material into the host genome, waiting for favourable conditions to induce cell lysis. During the lysogenic cycle, due to genome representation, the bacteriophage can lose its ability to replicate, and viral genes can be stably acquired by the host [150] and create the possibility of gene transfer.

It is believed that bacteriophage infection leads to the acquisition of tailocins and the VI secretion system by bacteria [151]. Tailocins are protein complexes similar in appearance to bacteriophage tails, which can recognise and attach to the neighbouring bacteria. The host is immune to the tailocins produced by itself thanks to the production of different lipopolysacharides that are targeted by the produced tailocin. These bacterial complexes tend to be specific to related bacteria, which supports their bacteriophage origin. Additionally, tailocins are often overproduced due to stress factors and realised by the burst of the host cell. Therefore, the tailocins are produced in the case of limited nutrient availability to avoid competition from closely related strains of bacteria [25]. The tailocins kill other cells by the depolarisation of their cell membrane. The tailocins, for example, were successfully used to control the *Nicotiana benthamiana* Domin. infection by *Pseudomonas syringae* [152].

The VI secretion system, similar to tailocins, is of bacteriophage origin. However, in this case, the protein complex is assembled in the cell membrane and does not lead to cell lysis. The assembled protein complex holds the kinetic energy, which is used to thrust the spike protein into the target neighbouring cell, either bacterial or eucariotic, perforating the cell membrane, leading to cell death. This system is used by microorganisms to compete with other microorganisms or by the pathogenicity of bacteria towards multicellular organisms [24]. The VI secretion system does not only thrust the spike into the cell, perforating it, but it can also be used to inject the toxin into the targeted cell cytoplasm, increasing the chance for its lysis.

Although the VI secretion system is a multiprotein complex, like tailocins or nanoscale particles, unlike them, they are not released into the medium and cannot be used as nanoparticles. However, the existence of these systems underlines that microorganisms produce and utilise nanoscale structures to interact with their environment, and the prevalence of the VI secretion system among different bacterial taxa emphasises its importance, indicating that this is a largely understudied topic in inter-microbial interactions.

## 5. Nanoencapsulation

The VI secretion system and the bacteriophages teach us that the nanoparticles can serve as a perfect carrier-delivery system. The size of the nanostructures can help the effective substance to reach the target, while their functionalisation with antibodies or oligonucleotides can guide them to the target. Thanks to this, nanoparticles can enhance the activity of the effector, minimalising the non-target toxicity [40]. Therefore, nanoencapsulation and nanoparticle delivery systems are promising technologies for decreasing the negative impact of the use of chemicals in agriculture [153]. What is more, the nano-encapsulated product shows superior shelf life in comparison to non-encapsulated products, further increasing the potential of this method [40].

### 5.1. Plant-Derived Extracellular Vesicles

Plants are able to protect themselves from pathogen invasion with the use of natural nanostructures: plant-derived extracellular vesicles. These extracellular vesicles are structures fragmented from the host surrounded by the host cell membrane harbouring cytoplasm and additional elements, such as proteins or RNAi. These vesicles can be used to deliver RNA to the pathogen to suppress its virulence [154]. Due to the size of these structures, they can be considered nanoparticles and are currently investigated for their potential of delivering vesicles in medicine [155]. Although it seems that the primary role of plant extracellular vesicles is physiological, it has been proven that they play an important role in plant defences against fungal and viral diseases [156]. The ability to deliver RNAi interfering in the plant pathogen virulence as well as effector proteins makes this structure a promising source of new methods of delivery of antimicrobials not only in plant protection but also applicable in human therapy [156]. The study on this delivery system has led to the discovery that extracellular vesicles play a major role in the interkingdom interactions between plants and pathogens [157].

The extracellular vesicles can not only provide a physical protection for carried substances but can also facilitate the transport of the carried effector substances through the cell membrane [154]. This feature can be used against the citrus canker pathogen *Xanthomonas axonopodis.* The effectiveness of biogenic silver nanoparticles against this important pathogen of citrus trees has been increased by their encapsulation in extracellular vesicles of *Phyllanthus niruri* [158]. On the other hand, the plant pathogens, such as *Pseudomonas syringae*, produce extracellular vesicles loaded with immunomodulatory proteins, which suppress *Arabidopsis thaliana* defences against this pathogen [159]. Furthermore, plant-beneficial microorganisms use extracellular vesicles for interkingdom communication, increasing plant fitness [160].

Extracellular vesicles, although in the upper limit of nanoparticle size, are a promising tool for the delivery of substances that can be used in agriculture, due to the facilitated penetration through the cell wall. However, many aspects of the interkingdom communication with extracellular vesicles are still unknown; it already shows promising results for the delivery of active substances [156].

### 5.2. Synthetic Nanocapsules

**Figure 3:**
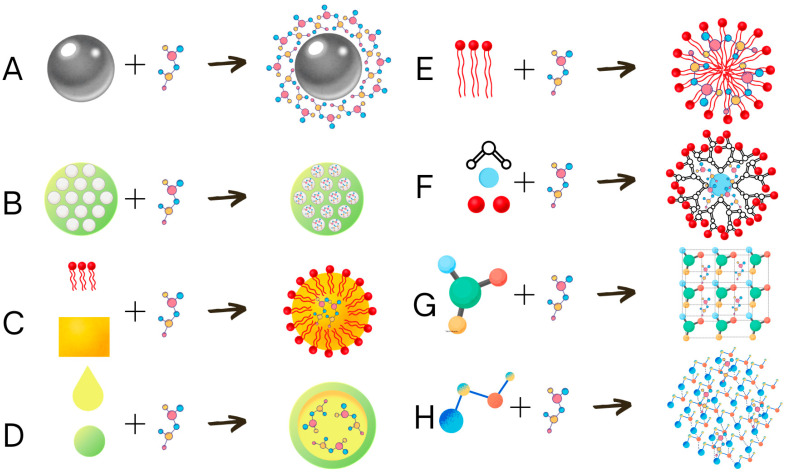
Schematic representation of nanoencapsulation types. The active substance can be delivered on the surface of metallic nanoparticles (**A**), inside the mesoporous silicon-based nanocarriers mesoporous (**B**), dissolved in solid lipid inside the solid lipid nanoparticles (**C**), dissolved in a liquid carrier inside the nanocapsules (**D**), carried inside micelles (**E**), carried by liquid-absorbing dendrimeric nanoparticles (**F**), incorporated into the crystal structure of nanocrystals (**G**), or trapped between the polymers of nanogels (**H**).

Nanoparticles can be used to deliver the effector substance to the target site by various methods. They can be transported on the surface of solid nanoparticles, such as metallic nanoparticles (Figure 3A), inside the mesoporous silicon-based nanocarriers (Figure 3B), solid lipid nanoparticles (Figure 3C), nanocapsules (Figure 3D), micelles (Figure 3E), dendrimers (Figure 3F), nanocrystals (Figure 3G), or nano gels (Figure 3H) [153]. Mesoporous silicon-based nanocarriers are built from mesoporous silicon material, with cavities that can be filled with the effector substance [161]. Solid lipid nanocarriers are produced by coating the liquid lipid matrix with dissolved effector substance by emulsifier [162]. Nanocapsules are formed by trapping the suspension of the effector in a polymeric capsule [163]. Micelles [164] and liposomes [165] can be spontaneously generated in hydrophilic conditions from amphiphilic substance surfactants in the case of micelles or lipid bilayers for liposomes. Dendrimers are spheres built from branched polymers branching from the phocal core, and the cavities between the branches can be used to carry the effector [166]. Nanocrystals can be formed by the crystallization of small crystals in the presence of an effector resulting in the formation of small crystal structures with incorporated effectors [167]. Nanogels are crosslinked polymers with a hydrophilic matrix containing effector molecules [153,168].

Although nanoencapsulation is often a complex process requiring strictly controlled conditions, it often requires the use of hydrophilic polymers. These substances can be synthetic, partially synthetic, or natural. These polysaccharides are useful for the production of nanogels, nanoemulsions, or nanocapsules, and bacteria are a rich source of polysaccharides with promising properties for creating nanoparticles [169]. Bacteria produce polysaccharides to build biofilm and protect themselves against desiccation or other physical or chemical factors [170]. The polysaccharides produced by microorganisms that find their application in the production of nanoparticles include inter alia: alginate, curldlan, dextran, gellan, levan, pullulan, and xanthan [169].

Microorganisms also produce a wide array of surfactants, which help them to maintain biofilm and attach to hydrophilic surfaces. This surfactant can find its application in agriculture by, for example, increasing the foliar absorption of nutrients, but also in the creation of micelles, which can be used for delivery of the effector molecules [171]. Microbial biosurfactants can not only be used for the production of micelles carrying plant protective effects but also possess bacteriocidal properties against many important fungal plant pathogens from genera: *Alternaria*, *Botrytis*, *Fusarium*, *Plasmopara*, *Phytophthora*, *Pythium*, and *Rhizoctonia* [171].

Although nanoencapsulation is widely studied for medical applications, agricultural applications will soon follow. The effectiveness of many therapeutics is limited due to their solubility and absorption through amphiphilic membranes. This problem can be addressed with the use of nanoparticles, which can guide the carried effector but also help it to cross the biological barriers. Similar problems occur in agriculture, and the already developed solution from pharmaceuticals can be applied in the production of plant protection products [153].

## 6. Conclusions

Nanoparticles are promising tools for the future development of not only medicine and technology but also for agriculture [153]. On the one hand, nanoparticles shelter biological substances from physical degradation, increasing the shelf life of nanoparticle-based products [40]. On the other hand, nanoparticles modified with antibodies or nucleotides can guide the carried effectors to the target site, reducing the amount of needed chemicals and decreasing the toxicity to the environment [40]. Although nanoparticles often possess antimicrobial properties that often show synergy with other chemicals, increasing their penetration to target organisms [93], the undeniable potential of nanoparticle application in the protection of plants against diseases lies in the creation of fast and robust pathogen detection methods that will help farmers quickly identify and eliminate the most devastating diseases [39]. Unfortunately, the production of nanoparticles often results in the production of large amounts of toxic waste [8]. This problem can, however, be tackled if we concentrate on the use of microorganisms for the production of nanoparticles or their components.

## Figures and Tables

**Table 1:** Nanoparticle-based detection methods for plant pathogens.

Nanoparticle	Modification	Detection Method	Pathogen	Host	Detection Limit	Reference
**AgNP**	antibodies	colorimetric	Potato Virus X (PVX)	Potato	2 ng/mL	Drygin et al., 2011 [41]
**AgNP**	antibodies	colorimetric	*Pantoea stewartii*	Corn	10^5^ cfu/mL	Feng et al., 2014 [42]
**AgNP**	antibodies	colorimetric	Banana bunchy top virus	Banana	32 ng/mL	Wei et al., 2014 [45]
**AgNP**	DNA	colorimetric	*Pseudomonas syringae*	-	15 µg/mL	Vaseghi et al., 2013 [46]
**CdSe QD**	DNA	FRET	*Ganoderma boninense*	-	3.55 µMol/mL	Bakhori et al., 2013 [47]
**CdTe** **QD**	antibodies	FRET	Candidatus Phytoplasma aurantifoli	-	5 × 10^3^ cfu/mL	Rad et al., 2012 [48]
**CdTe QD**	antibodies	FRET	Citrus tristeza virus (CTV)	Citrus trees	220 ng/mL	Shojaei et al., 2016 [49]
**CdTe QD**	antibodies	FRET fluorescence	Citrus Tristeza Virus (CTV)	Citrus trees	198 ng/mL 246 ng/mL	Safarnejad et al., 2017 [50]
**CdTe QD**	antibodies	FRET	*Polymyxa betae*	Sugarbeet	0.5 µg/mL	Safarpour et al., 2017 [51]

AgNp silver nanoparticle; CdSe Cadmium Selenide; QD Quantum Dot; CdTe QD Cadmium Tellurite Quantum Dot; FRET fluorescence resonance energy transfer.

**Table 2:** Microbial nanoparticles used to protect plants from important plant pathogens.

Producer	Nanoparticle	Pathogen	Reference
*A. niger*	AgNP	*Aspergillus terreus*, *F. oxysporum*, *Penicillium citrinum*, *Rhizopus stolonifera*, and *Mucor mucedo*	Ahmad et al., 2024 [101]
*Acinetobacter johnsonii*	MgONP	*Acidovorax oryzae*	Ahmed et al., 2021 [102]
*Aeromonas* *hydrophila*	ZnONP	*Pseudomonas aeruginosa*, *Aspergillus flavus*	Jayaseelan et al., 2012 [103]
*Aspergillus* *flavus*	AgNP	*A. terreus*, *F. oxysporum*, *P. citrinum*, *R. stolonifera*, and *M. mucedo*	Ahmad et al., 2024 [101]
*Bacillus* *cereus*	AgNPs	*Xanthomonas oryzae*	Ahmed et al., 2020 [97]
*Bacillus* *megaterium*	MnNP	*F. oxysporum*	Noman et al., 2022 [104]
*Bacillus* *altitudinis*	CuNP	*Acidovorax citrulli*	Noman et al., 2023 [105]
*Bacillus* *aryabhattai*	BNCs	*X. oryzae*	Ahmed et al., 2022 [106]
*Burkholderia* *cepacia*	AgNPs	*A. niger, A. fumigatus*, *F. oxysporum*, *Pythium* sp., and *Rosellinia* sp.	Mittal et al., 2022 [107]
*Enterobacter* sp.	ZrONP	*Pestalotiopsis versicolor*	Ahmed et al., 2021 [108]
*Paenibacillus* *polymyxa*	ZnONP, MnO_2_NP, and MgONP	*X. oryzae*	Ogunyemi et al., 2020 [109]
*Pencillium* *chrysogenum*	AgNP	*A. terreus*, *F. oxysporum*, *P. citrinum*, *R. stolonifera*, and *M. mucedo*	Ahmad et al., 2024 [101]
*Pseudomonas* *rhodesiae*	AgNPs	*Dickeya dadantii*	Hossain et al., 2019 [96]
*Serratia* *marcescens*	AgNPs	*Aspergillus niger*, *A. fumigatus*, *Fusarium oxysporum*, *Pythium* sp., and *Rosellinia* sp.	Mittal et al., 2022 [107]
*Streptomyces* *zaomyceticusand*	CuONP	*F. oxysporum*, *Pythium ultimum*, *A. niger* and *Alternaria alternata*	Hassan et al., 2019 [110]
*Streptomyces griseus*	CuNP	*Poria hypolateritia*	Ponmurugan et al., 2016 [111]
*Streptomyces pseudogriseolus*	CuONP	*F. oxysporum*, *Pythium ultimum*, *A. niger* and *Alternaria alternata*	Hassan et al., 2019 [110]

AgNP silver nanoparticle, MnNP manganese nanoparticles, CuNP copper nanoparticles, BNCs bioengineered chitosan iron nanocomposites, ZnONP zinc oxide nanoparticles, MnO_2_NP manganese oxide nanoparticles, MgONP magnesium oxide nanoparticles, ZrONP zirconium oxide nanoparticles.

## Data Availability

Data sharing is not applicable to this article.
